# Brain Structural Features of Myotonic Dystrophy Type 1 and their Relationship with CTG Repeats

**DOI:** 10.3233/JND-190397

**Published:** 2019

**Authors:** Ellen van der Plas, Mark J. Hamilton, Jacob N. Miller, Timothy R. Koscik, Jeffrey D. Long, Sarah Cumming, Julija Povilaikaite, Maria Elena Farrugia, John McLean, Ravi Jampana, Vincent A. Magnotta, Laurie Gutmann, Darren G. Monckton, Peggy C. Nopoulos

**Affiliations:** a Department of Psychiatry, University of Iowa Hospital and Clinics, Iowa City, IA, USA; b West of Scotland Clinical Genetics Service, Queen Elizabeth University Hospital, Glasgow, UK; c Institute of Molecular, Cell and Systems Biology, College of Medical, Veterinary and Life Sciences, University of Glasgow, Glasgow, UK; d Department of Biostatistics, University of Iowa, College of Public Health, Iowa City, IA, USA; e Department of Neurology, Institute of Neurological Sciences, Queen Elizabeth University Hospital, Glasgow, UK; f Department of Neuroradiology, Institute of Neurological Sciences, Queen Elizabeth University Hospital, Glasgow, UK; g Department of Radiology, University of Iowa Hospital and Clinics, Iowa City, IA, USA; h Department of Neurology, University of Iowa Hospital and Clinics, Iowa City, IA, USA

**Keywords:** Myotonic dystrophy, magnetic resonance imaging, neuroanatomy

## Abstract

**Background:**

Few adequately-powered studies have systematically evaluated brain morphology in adult-onset myotonic dystrophy type 1 (DM1).

**Objective:**

The goal of the present study was to determine structural brain differences between individuals with and without adult-onset DM1 in a multi-site, case-controlled cohort. We also explored correlations between brain structure and CTG repeat length.

**Methods:**

Neuroimaging data was acquired in 58 unaffected individuals (29 women) and 79 individuals with DM1 (50 women). CTG repeat length, expressed as estimated progenitor allele length (ePAL), was determined by small pool PCR. Statistical models were adjusted for age, sex, site, and intracranial volume (ICV).

**Results:**

ICV was reduced in DM1 subjects compared with controls. Accounting for the difference in ICV, the DM1 group exhibited smaller volume in frontal grey and white matter, parietal grey matter as well as smaller volume of the corpus callosum, thalamus, putamen, and accumbens. In contrast, volumes of the hippocampus and amygdala were significantly larger in DM1. Greater ePAL was associated with lower volumes of the putamen, occipital grey matter, and thalamus. A positive ePAL association was observed for amygdala volume and cerebellar white matter.

**Conclusions:**

Smaller ICV may be a marker of aberrant neurodevelopment in adult-onset DM1. Volumetric analysis revealed morphological differences, some associated with CTG repeat length, in structures with plausible links to key DM1 symptoms including cognitive deficits and excessive daytime somnolence. These data offer further insights into the basis of CNS disease in DM1, and highlight avenues for further work to identify therapeutic targets and imaging biomarkers.

## INTRODUCTION

Myotonic dystrophy type 1 (DM1) is a dominantly inherited, multisystem disorder, resulting from expansion of a CTG trinucleotide repeat in the 3’-untranslated region of *DMPK* [1]. The clinical phenotype is widely variable, with common symptoms including skeletal muscle weakness and myotonia, cataracts, cardiac conduction defects, and male hypogonadism. Involvement of the CNS underlies additional symptoms such as cognitive deficits, excessive daytime somnolence, apathy, and impaired social functioning [2]. CNS symptoms are reported frequently by patients [3], and may greatly impact quality of life [4, 5], relationships with caregivers [6] and engagement with medical care [7]. With the advent of clinical trials of potential disease-modifying therapies for DM1, expert working groups have highlighted the need to improve understanding of the mechanisms underlying CNS disease, both to identify neuroimaging biomarkers and reveal potential targets for therapy [8, 9].

MRI studies in patients with DM1 have identified morphometric abnormalities in the brain, including global volume loss, ventricular dilation, white matter lesions, and disruptions of white matter microstructure evident in diffusion tensor imaging [10]. According to several studies that used voxel-based morphometry (VBM), global volume loss may be driven by grey matter atrophy in the cerebral cortex and subcortical structures [11–13]. Some VBM studies have also demonstrated associations between focal brain morphology and cognitive abilities [14, 15], and/or CTG repeat length [16, 17], although others found no association [12, 13].

Several limitations of preceding neuroimaging studies should be considered. First, previous studies have generally included fewer than 40 affected individuals, limiting the power to detect meaningful abnormalities in brain morphology [11, 13, 15, 18, 19]. Second, few studies have validated the VBM findings with alternative approaches that enable comprehensive exploration of global and regional grey and white matter structural differences between patients and controls [18]. Third, previous studies have relied on traditional methods to measure CTG repeat expansion which fail to take account of age-dependent, expansion-biased mosaicism in somatic cells [20]. Finally, some existing studies have combined congenital DM1 (CDM), juvenile-onset DM1 (JDM), and adult-onset DM1 in a single sample [13, 17]. Individuals with CDM exhibit severe cognitive delay [21], while those with adult-onset DM1 typically have much milder impairment [22]. The diversity of CNS phenotypes in heterogeneous samples confounds analyses of structural correlations further.

To address these limitations, we harmonized neuroimaging protocols across two sites to obtain a large, homogeneous, case-controlled sample of individuals with adult-onset DM1. A volume-based brain parcellation approach was applied to characterize brain structures. Genotyping was conducted by small-pool PCR (SP-PCR) to estimate the progenitor allele length, which has been shown to improve clinical correlations compared to traditional methods [23].

## MATERIALS AND METHODS

### Participants

Individuals with DM1 were recruited to the University of Iowa from across the United States via advertisements through the Myotonic Dystrophy Foundation (MDF) and word of mouth. Unaffected participants were primarily recruited from the Iowa City area via advertisements. Some were unaffected spouses of affected participants. Recruitment took place between September 2014 and July 2017.

In Glasgow, UK, individuals with DM1 attending the annual review at the West of Scotland Clinical Genetics Service were invited to participate. Unaffected participants were recruited from affected subjects’ families and from the Scottish Health Research Register(www.registerforshare.org) [24]. Participants in Glasgow were recruited between January 2016 and May 2017.

Exclusion criteria for all participants were: learning disability in childhood, a history of serious head injury, or a chronic neurological disorder other than DM1. Unaffected participants were additionally required to be without history of substance abuse, psychiatric disease, or major medical disease, including: heart disease, sleep disorder, vascular disease, uncontrolled hypertension, cancer, diabetes mellitus, lung disease, and autoimmune conditions.

Recruitment was targeted to adult-onsetDM1only. The Glasgow site recruited only patients who denied onset of DM1-specific symptoms before age 16, while the Iowa site included participants who denied symptoms before age 18. The sample included seven individuals who had self-reported age of first onset before age 18.

Participants underwent genetic testing as part of the research study. All participants recruited into the DM1-affected group at the Glasgow site, and the majority at the Iowa site (93%), had also undergone genetic testing prior to participation and were aware of their genetic status. A subset of participants in Iowa were at-risk for DM1, but had not undergone predictive testing (*n* = 11). At-risk individuals in the Iowa sample who were determined to have CTG repeat length >50 were included in the DM1 group (*n* = 4). The remainder had CTG repeat lengths in the non-disease associated range, and were included in the control group (*n* = 7). Research staff, clinicians and scientists involved in this study remained blind to the genetic status of at-risk individuals. All data were de-identified and all participants at the Iowa site consented to non-disclosure of genetic results obtained as part of the study. Control participants recruited in Glasgow were not genotyped for CTG repeat length, but none was at risk of DM1 by family history, and none exhibited overt signs of any neurological disorder. Control participants in Iowa were genotyped for CTG repeat length, to confirm unaffected status.

All participants gave written, informed consent prior to enrolling in the protocol in accordance to the Declaration of Helsinki. The study was approved by the University of Iowa’s Institutional Review Board or the West of Scotland Research Ethics Committee (Reference: WOS 15/WS/0189).

### Muscle impairment rating scale (MIRS)

Severity of muscle weakness, expressed as muscle impairment rating scale (MIRS) [25],was determined by examination by a neurologist or other specialist experienced in DM1.

### Image acquisition

Iowa participants who participated before June 2016 (*n* = 52) were scanned using a 3T Siemens Trio TIM (Siemens AG, Munich, Germany; 12-channel head coil), and those who participated after June 2016 (*n* = 27) were scanned on 3T General Electric Discovery MR750w (GE Medical Systems, Chicago, IL, 16-channel head and neck coil). Anatomical T1-weighted images were acquired as follows for Siemens (GE parameters in parentheses): coronal MPRAGE (BRAVO), TR = 2300 (8.392) ms, TE = 2.82 (3.184) ms, TI = 900 (450) ms, flip angle = 10 (12)º, FOV = 282 × 282 × 264 mm, matrix = 256 × 256 × 240. Parameters for T2-weighted images were: coronal, TR = 4800 (3000) ms, TE = 430 (85.925) ms, FOV = 256 × 256 × 224 mm, matrix = 256 × 256 × 160.

In Scotland, imaging was carried out on a 3T Siemens Prisma MRI scanner (Software version: VE11B. Erlangen, Germany, 20-channel head and neck coil). T1-weighted parameters: coronal MPR AGE, TR = 2300 ms, TE = 2.01 ms, TI = 900 ms, flip angle = 10º, FOV = 282 × 282 × 211 mm, matrix = 256 × 256 × 192. T2-weighted parameters coronal SPACE, TR = 3200 ms, TE = 407 ms, FOV = 282 × 198 × 194 mm, matrix = 256 × 180 × 176.

### Image processing

Bias field inhomogeneity was corrected using the N4 algorithm implemented in Advanced Normalization Tools software [26]. Images were processed using the BRAINSAutoWorkup pipeline which optimizes tissue classification through an iterative framework and produces robust parcellation of brain regions in a multi-site setting [27]. BRAINSAutoWorkup labels brain regions using a multi-atlas, similarity-weighted, majority-vote procedure (joint label fusion, [28]) using a set of expert-segmented templates adapted from the Desikan-Killiany atlas [29]. Brain regions include cortical and subcortical regions, separated by hemispheres and tissue type (gray or white matter) where appropriate. Residual inter-scanner variation was harmonized using an empirical Bayesian approach [30, 31] as implemented by the ez.combat toolbox in R [32]. We confirmed that scanner did not predict regional volume by conducting Kolmogorov-Smirnov tests (Supplementary Table 1) and visualizing the empirical cumulative distributions for each scanner across ROIs and groups (Supplementary Figure 1). Statistical analyses were performed on the harmonized neuroimaging data.

### Measurement of CTG repeat length

Genotyping of CTG repeat in DM1 participants was completed by SP-PCR [33]. For each patient, four reactions were completed, each using 300 pg blood genomic DNA template. CTG repeat lengths were estimated by comparison against DNA fragments of known length in the molecular weight marker, using CLIQS software (TotalLabs UK Ltd.). The lower boundary of the expanded molecules in SP-PCR was used to estimate the inherited or progenitor allele length (ePAL) [34], which is the major determinant of age at symptom onset [23].

### Statistical analyses

It is important to account for intracranial volume (ICV) when comparing regions between groups, because regions scale with ICV. The goal of correction is to transform the region of interest (ROI) such that it is no longer related to ICV, which requires accounting for non-linear relationships. The power-proportion method (PPM) divides volume by β, where β is estimated from a non-linear regression model, *ROI* = α*ICV*^β^ [35]. We estimated β for each ROI, and divided ROI by ICV^β^. Each ratio was subsequently standardized by subtracting out the grand mean, and dividing by the SD. The efficacy of detrending was checked by running linear regression models predicting the adjusted ROI from ICV. None of the estimates were statistically significant (unadjusted, lowest *p*-value = 0.647; see Supplementary Figure 2).

The multivariable linear regression models to examine group differences included the harmonized, standardized, PPM-adjusted ROI volumes as the dependent variable, and group, age, sex, and site as predictors:

$$Z_{i}=\gamma_{0}+\gamma_{1}{\text{ group}}_{i}+\gamma_{2}{\text{ age}}_{i}+\gamma_{3}{\mathrm{sex}}_{i}+\gamma_{4}{\text{ site}}_{i}+e_{i},$$

In this equation, Z_*i*_ is the adjusted ROI volume for the *i*th participant (*I* = 1, *. . .*, *N*). Group, sex, and site were expressed as numeric factors. It was assumed that the *e*_*i*_ were normally distributed with zero mean and non-zero variance.

The models for ePAL considered only the DM1 group:

$$Z_{i}=\gamma_{0}+\gamma_{1}\;ePAL_{i}+\gamma_{2}\;age_{l}+\gamma_{3}\;sex_{i}+\gamma_{4}\;site_{i}+e_{i},$$

Group and ePAL estimates are presented as adjusted means normalized to the standard deviation. Inference was based on the 99% confidence interval (CI).

## RESULTS

### Sample

**Fig. 1.**
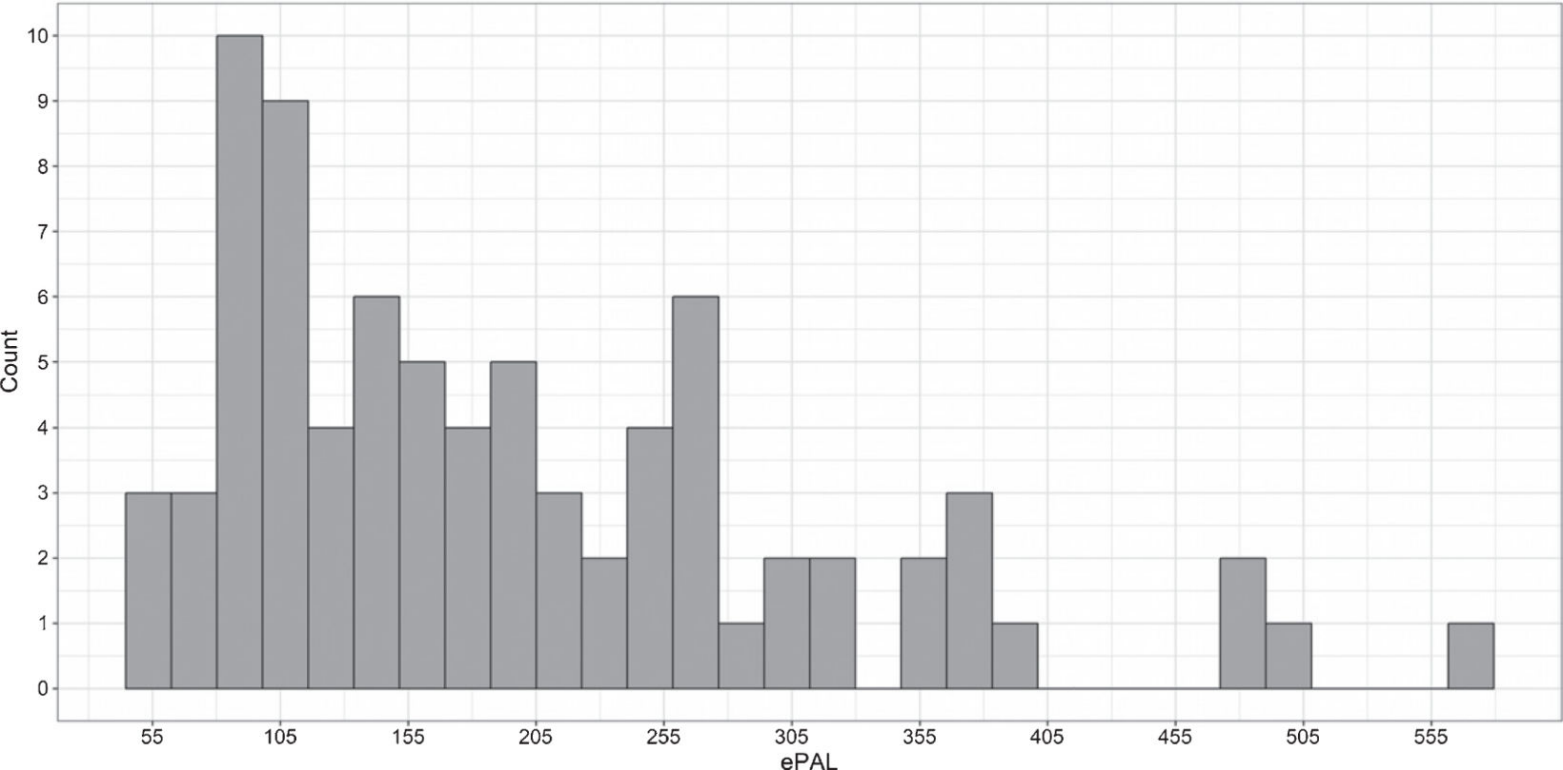
Distribution of CTG repeat length (expressed as ePAL) among individuals with DM1 in the sample.

The pooled sample included 58 unaffected individuals and 79 individuals with DM1. The Iowa site contributed 38 unaffected individuals and 41 individuals with DM1, and the Glasgow site contributed 20 unaffected individuals and 38 individuals with DM1 (Table 1). The unaffected group included equal numbers of men (*n* = 29) and women (*n* = 29), while the DM1 group included significantly more women (*n* = 50) than men (*n* = 29), *χ*^2^_(1)_ = 6.0, *p* = 0.02. Note that statistical models are adjusted for sex. Average age at evaluation was 46 years old (SD = 13 years), with no significant difference between groups, *t*_(134)_ = 0.5, *p* = 0.6. ePAL values in the DM1 group ranged from 55 to 572 CTG repeats (Fig. 1), and self-reported age at onset of DM1 symptoms was on average 33 years old (SD = 12 years) (Table 1).

### Brain morphology

**Fig. 2.**
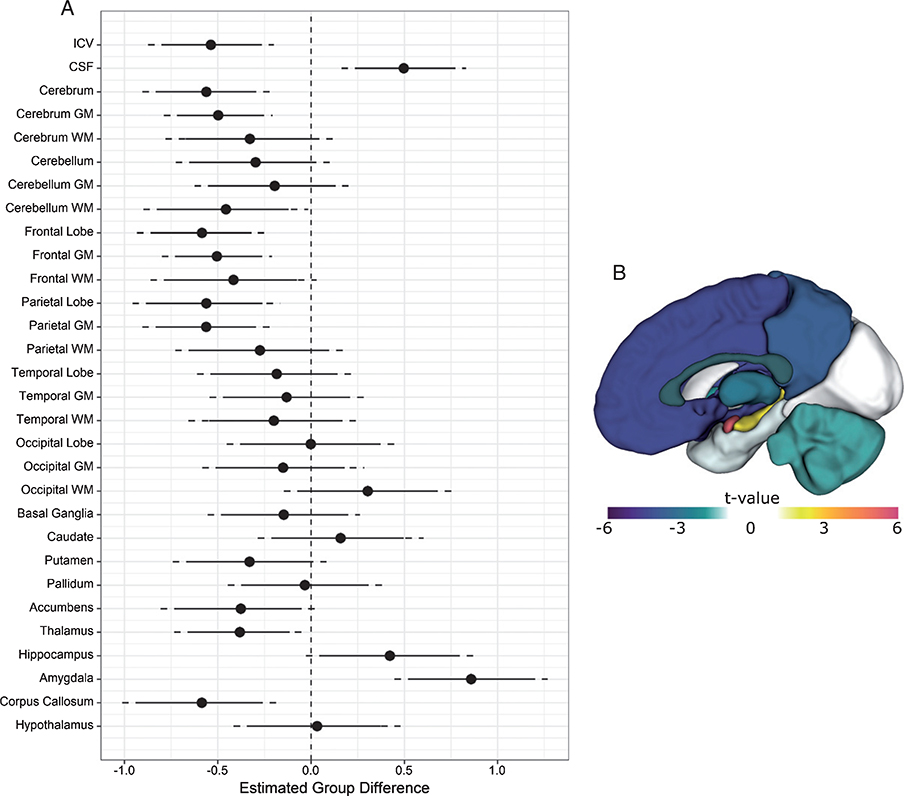
Structural brain differences between DM1 patients and unaffected adults. Panel A: The circles represent estimated group differences adjusted for sex, age and site in SD units (x-axis) for regional volume (y-axis). The vertical, dashed line marks 0, i.e., no group difference. The solid lines represent the 95% confidence limits, and the dashed lines the 99% confidence limits. Negative differences indicate that the DM1 group had smaller volumes than did controls, while positive differences indicate that the DM1 group had larger volumes than controls. Panel B: Summary of group differences in regional volumes between individuals with and without DM1 after ICV adjustment. The colors correspond with the magnitude of the *t*-values of group differences (see color scale). Cool colors indicate that the DM1 group had lower volume than the unaffected group, while hot colors indicate the opposite pattern.

Sex was a significant predictor of intracranial volume (ICV), with women exhibiting lower ICV than men, *t*_(132)_ = 9.6, *p* < 0.0001. Age did not predict ICV (*p* = 0.1). The group coefficient for ICV was significant (*t*_(132)_ = −4.1, *p* < 0.0001), with mean ICV being 7.6% lower in the DM1 group compared with unaffected individuals (Fig. 2A).

Both sex and age were significant predictors of cerebrospinal fluid volume, *t*_(132)_ = 2.8, *p* < 0.01, and *t*_(132)_ = 9.6, *p* < 0.00001, respectively. The group coefficient was also statistically significant (*t*_(132)_ = 3.8, *p* < 0.001). Individuals affected with DM1 had approximately 11% greater CSF volume than unaffected individuals (Fig. 2A).

Compared with unaffected individuals, the DM1-affected group exhibited significantly lower cerebral volume (*t*_(132)_ = −4.2, *p* < .0001), evidently driven mostly by cerebral grey matter (*t*_(132)_ = −4.4, *p* < 0.0001). Cerebellar white matter was significantly reduced in DM1 as well, *t*_(132)_ = −2.7, *p* = 0.01. Across cerebral lobes, the DM1 group exhibited lower volume in frontal grey (*t*_(132)_ = −4.4, *p* < 0.0001) and white matter (*t*_(132)_ = −2.4, *p* = 0.02), parietal grey matter (*t*_(132)_ = −4.2, *p* < 0.0001), as well as corpus callosum (*t*_(132)_ = −3.5, *p* < 0.0001). Subcortically, affected individuals exhibited lower volumes of putamen (*t*_(132)_ = −2.1, *p* = 0.04), accumbens (*t*_(132)_ = −2.3, *p* = 0.03), and thalamus (*t*_(132)_ = −2.8, *p* = 0.01). By contrast, hippocampus (*t*_(132)_ = 2.4, *p* = 0.02) and amygdala volume (*t*_(132)_ = 5.4, *p* < 0.00001) were larger in individuals affected with DM1 (Fig. 2A and 2B). Unadjusted parcellation volumes in these regions also appeared larger in the DM1-affected group prior to standardization (Supplementary Figure 3).

**Fig. 3.**
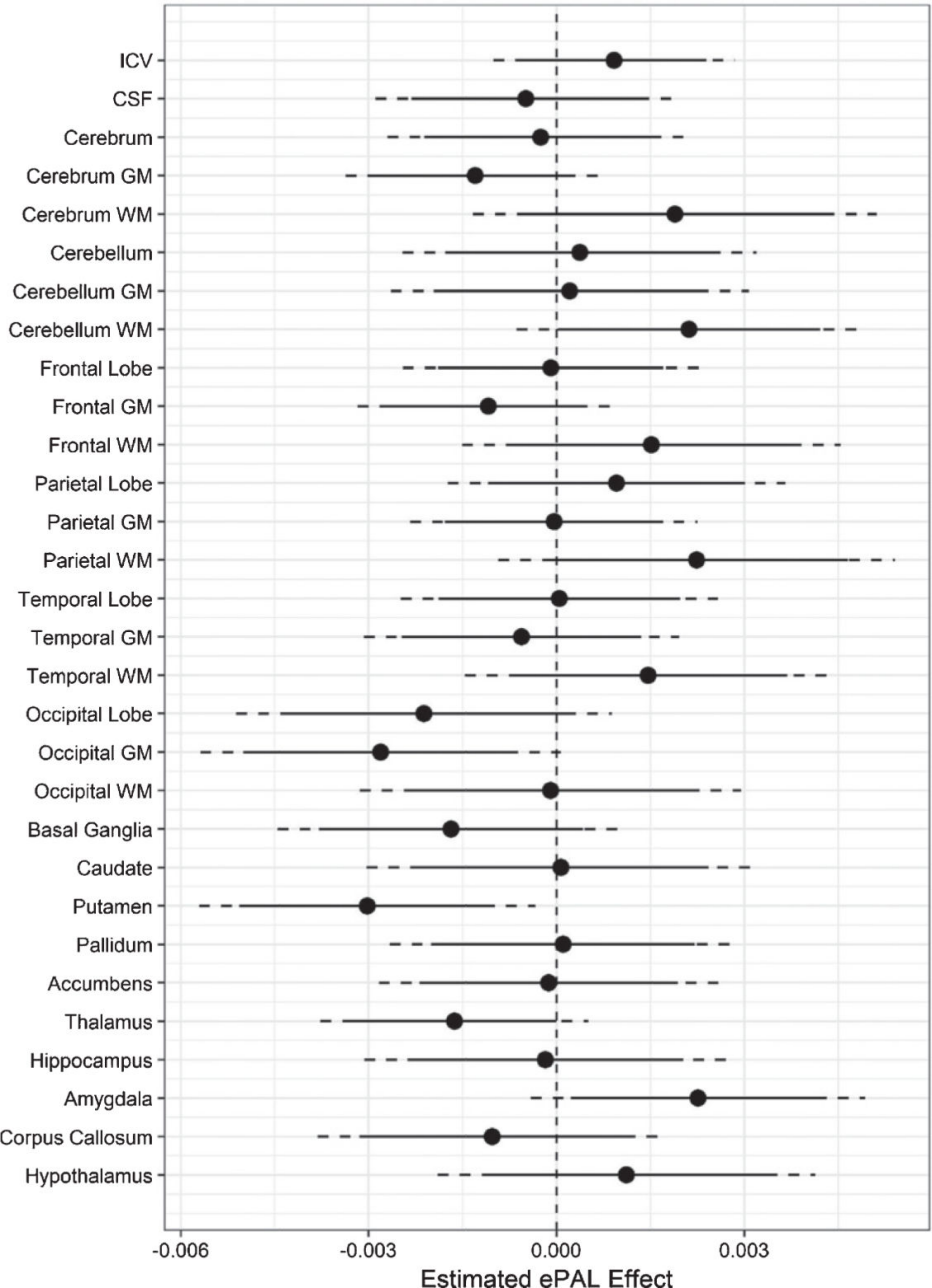
Association between regional brain volume and ePAL in DM1. The circles represent estimated coefficients (circles) for the relationship between ePAL and regional volume (y-axis), adjusted for sex, age, and site in SD units (x-axis) among individuals with DM1. The vertical, dashed lines marks 0, i.e., no effect of ePAL. The solid lines represent 95% confidence limits, and the dashed lines represent 99% confidence limits.

Analyses indicated a negative coefficient for ePAL when predicting adjusted putamen volume (*t*_(132)_ = −2.9, *p* = 0.01), occipital grey matter (*t*_(132)_ = −2.5, *p* = 0.01), and thalamus (*t*_(132)_ = −2.0, *p* = 0.05; Fig. 3). A positive ePAL coefficient was observed for amygdala volume (*t*_(132)_ = 2.2, *p* = 0.03), and cerebellar white matter (*t*_(132)_ = 1.98, *p* = 0.05) (Fig. 3).

## DISCUSSION

The present study constitutes the largest neuroimaging study in DM1 to date, demonstrating that multi–site neuroimaging is a feasible, powerful approach to elucidate mechanisms of CNS disease in DM1. We restricted our sample to adult-onset DM1 to limit confounders associated with the markedly diverse clinical continuum, such as intellectual deficits that are evident in congenital and juvenile onset DM1 [21]. We also added a comprehensive, volumetric parcellation approach to the existing literature on brain morphology in DM1, and identified regional structural brain abnormalities in individuals with adult-onset DM1. We demonstrated that variation in volume of the putamen, occipital gray matter, thalamus, and amygdala was significantly predicted by CTG repeat expansion, strengthening the link between regional abnormalities and disease-specificity.

In line with earlier work [15, 36–39], we replicate evidence of lower ICV in individuals with DM1. ICV is considered a marker of maximal brain growth and is largely determined by the age of 10 years, irrespective of subsequent changes in the brain parenchyma [40]. The finding of reduced ICV is therefore particularly notable in our cohort, in which recruitment was targeted to the adult-onset form of DM1. While it must be acknowledged that timing of onset of symptoms is a somewhat subjective measure, and it cannot be excluded that some subjects could have manifested subtle features ofDM1in childhood, this finding does imply that presence of the expanded CTG repeat has an effect on CNS structure prior to the onset of overt classical DM1 symptoms.

The cause of restricted brain volume in DM1 is not known. It could be hypothesised to result from a variety of mechanisms, including abnormal development, differences in cell volume, reduced proliferation, or early loss of neurons [41, 42]. In patients with schizophrenia, who show more subtle reduction in ICV [43], reduced grey matter volume and synaptic density is believed to occur due to an excess of synaptic pruning [44], the physiological process of neural remodelling that typically occurs from childhood through adolescence. Early-onset, excessive pruning could conceivably represent an additional mechanism affecting maximum ICV growth in DM1. Of note, it is unclear whether the modest reduction of IQ observed in cohorts with adult-onset DM1 entirely represents a neurodegenerative process [45], or may have its roots in abnormal brain development. Neurodevelopmental aspects of adult-onset DM1 therefore represent a key area for further work to improve understanding of CNS disease.

Regional brain volume was found to be reduced in DM1-affected subjects, while CSF volume was greater in the DM1 group than the unaffected group. Studies on the aging brain or in neurodegenerative disorders have demonstrated that atrophy coincides with increased ventricular spaces and greater CSF volume [46, 47]. Regional volume reductions inDM1 are thought to be the result of atrophy [10, 19], therefore the observed increase in CSF is to be expected. We observed reduced volume of frontal and parietal lobes, as well as substantial loss in subcortical structures including thalamus and putamen, consistent with the findings of previous studies in smaller cohorts using alternative neuroimaging techniques [11–13, 18]. The functional impact of structural changes observed is not clear from the available data, so the need to explore clinical correlations of brain morphological abnormalities remains. The functional impact of subcortical volume loss is of particular interest, given the roles of these structures in maintaining wakefulness, regulating sleep architecture, and in cognitive processing [48], which could clearly be relevant to elements of the DM1 phenotype.

Variation in putamen, thalamus and occipital grey matter volume was negatively related to the size of the CTG repeat expansion (adjusting for covariates), while amygdala volume was positively associated with CTG repeat length. These results support a direct association between these regional brain abnormalities and the primary genetic lesion in DM1. The positive association between CTG repeat length and cerebellar white matter volume is somewhat puzzling, given the observation that cerebellar white matter was significantly *reduced* in the DM1 group compared with the unaffected group.

The processes by which CTG expansion leads to structural brain change in DM1 are incompletely understood. Histopathology studies of brain suggest a complex phenotype, including widespread neuronal loss, presence of neurofibrillary tangles, hyalinisation of capillary walls and diffuse widening of perivascular spaces [49, 50]. At the cellular level, CUG repeat mRNAs arising from the expanded repeat are seen to form discrete foci in the nuclei of DM1-affected neurons. Presence of these foci is associated with a dysregulation of alternative splicing affecting a wide range of mRNA targets [51], with the potential to impact a range of critical pathways and structural proteins in neural cells. For example, tau protein is subject to a shift in alternative splicing, favoring fetal isoforms [52]. These isoforms have lower affinity to bind and stabilize the microtubular cytoskeleton, and may disrupt axonal transport. Overlapping histological features between DM1 and Alzheimer dementia, including presence of neurofibrillary tangles, strongly supports a role of tau dysfunction in CNS pathogenesis [53]. Evidence for dysregulated splicing of the NMDA receptor subunit *NMDAR1* [54] and altered expression of synaptic vesicle proteins [55] suggest a role for disruption of synaptic signalling also.

It is possible that different mechanisms are predominantly responsible for driving structural change in different regions of brain, which could account for variation in the degree of volumetric change and strength/direction of correlations with the CTG repeat between regions. Expansion-biased somatic instability of the CTG repeat in DM1 varies between tissues, and is generally seen to be greater in the tissues most affected by the disease, such as muscle [56]. Intriguingly, in the CNS, somatic instability is comparatively low in cerebellar cortex compared with frontal cerebral cortex [50], consistent with the relative severity of volume loss observed in these structures. Further studies of postmortem brain would be useful to gather further evidence for variation in pathology in different brain regions. Systematic measurement of somatic instability in specific structures would be particularly useful to evaluate this mechanism as a driver of CNS pathology, and hence possible target for therapy.

Larger volume of amygdala and hippocampus was an unexpected finding, particularly in the context of widespread volume loss. This observation is notable in the context of a previous study using VBM which reported reduced hippocampal volume in DM1 [39]. In addition to using a different parcellation approach, the present study included a sample around five times larger than the study by Weber and colleagues. It should also be noted that the observed increase in amygdala and hippocampal volumes was not an artefact of our standardization method, as the DM1 group exhibited larger *unadjusted* volumes in these regions than unaffected controls (see Supplementary Figure 3).

The cause of increased volume is unclear, but may relate to loss of mechanical growth restriction due to atrophy of adjacent structures, a compensatory change to preserve function in neuronal systems, abnormal growth driven by the molecular pathology of DM1, a combination of these factors, or other processes. The limited histological evidence available from previous studies suggest amygdala and hippocampus are not spared from the disease process, in that they exhibit typical hallmarks of DM1 including neurofibrillary tangles [49, 57]. Interestingly, sleep disordered breathing has been linked to both regional increases and decreases in hippocampus volume in non-DM1 subjects, held to represent the acute and chronic effects of hypoxia respectively [58]. Further, emerging evidence suggests a functional link between amygdala and control of respiration, with stimulation of the amygdala seen to induce apnea [59]. Given the high prevalence of sleep disordered breathing in DM1, including presence of central apneas [60], clinical correlations with respiratory measures would be important to explore in future work. The amygdalo-striatal circuitry has further been implicated in motivational salience [61], raising questions about the potential role of amygdalo-striatal dysfunction in symptoms of depression, apathy, and anhedonia that are commonly observed among individuals with DM1 [62]. Current efforts to develop therapy for anxiety disorders, through manipulation of specific inhibitory neuron populations within the amygdala [63], could therefore yield useful therapeutic targets for a range of CNS symptoms in DM1.

A limitation of the present study is that volumetric analysis gives limited information regarding the structural or functional integrity of white matter. In contrast to some previous data [11], we found that volumetric changes were generally less marked in white matter structures compared with associated grey matter. Nonetheless, white matter pathology clearly represents an important component of the CNS structural landscape in DM1, as evidenced by the frequent occurrence of T2 hyperintense lesions [10], and widespread reduced fractional anisotropy on diffusion tensor imaging, even in normal-appearing white matter [64]. It remains uncertain to what extent white matter disease represents a primary manifestation of the DM1 disease process, versus Wallerian degeneration secondary to loss of associated grey matter [16]. Given that a number of disease processes likely conspire to cause structural brain changes in DM1, it is clear that a range of modalities should be employed in future neuroimaging studies. Future studies would also benefit from a longitudinal design. Of note, recent data from Gliem and colleagues [65] suggest the rate of structural brain change over time may be slow, and hence could be challenging to measure within the timescale of a typical clinical trial. Further, respiratory function was not specifically assessed as part of the present study. Sleep-disordered breathing is common in DM1 [66], and has been linked to various structural brain changes in other patient populations [58, 67, 68], therefore detailed phenotyping of respiratory function including sleep studies should be considered as part of future neuroimaging work. Finally, the clinical impact of the structural changes identified were not explored as part of the present study. Correlation of structural measures with neuropsychological data would be an important extension of the work presented here, to identify how abnormal brain structure may relate to functional problems.

In summary, the present study highlights the potential for multi-site neuroimaging efforts to elucidate neuropathological features associated with inherited, rare neurological disorders, such as DM1. The pooled sample represents the largest case-control DM1 neuroimaging cohort at present. The study provided novel insights into the effects of DM1 on the human brain, by providing a detailed portrait of the relative volumetric changes occurring in specific regions. Several of the structural changes were directly correlated with CTG repeat length, and occurred in structures with potential functional links to key features of the DM1 phenotype. These findings illuminate potential avenues for further research work to elucidate the molecular and neuroanatomical basis for central symptoms in DM1.

## Supplementary Material

Supplementary Table 3

Supplementary Table 2

Supplementary Table 1

Supplementary Figure 1

Supplementary Figure 2

Supplementary Figure 3

## Figures and Tables

**Table 1:** Site characteristics

			Controls		DM1	
			Scotland	Iowa	Scotland	Iowa
Sample	N		20	38	38	41
Sex	N	Males	12	17	17	12
		Females	8	21	21	29
Age at evaluation		Mean (SD)	46.1 (13.1)	44.6 (13.7)	47.1 (13.2)	45.4 (11.7)
Age at symptom onset		Mean (SD)	–	–	30.5 (13.4)	35.4 (10.3)
Muscle impairment	N	MIRS 1	–	–	3	12
		MIRS 2	–	–	6	19
		MIRS 3	–	–	10	5
		MIRS 4	–	–	18	5
		MIRS 5	–	–	1	0
ePAL		Range	–	–	56–572	55–501
		Median	–	–	211	131
		Interquartile range	–	–	152–269	88–215
