# Supplementary material for: Brain Structural Features of Myotonic Dystrophy Type 1 and their Relationship with CTG Repeats
Source: J Neuromuscul Dis. Author manuscript; Available in PMC 2020 Sep 9. (PMC7480174; doi:10.3233/JND-190397)
Supplement: Supplementary Table 3 [file NIHMS1623355-supplement-Supplementary_Table_3.pdf]

Supplemental Table 3. Effect Sizes for Table 2 in the Main Document.

Table 3

Between Group Effect Sizes

Subscale	Noncentrality Parameter MVUE	Noncentrality Parameter Low MSE E	Noncentrality Parameter 95% CI2	Total Variation Accounted For η^2	Total Variation Accounted For Ω^2	Total Variation Accounted For 95% CI[2]
BIS-11 Nonplanning	20.4	20.2	6.3 - 45	0.1023	0.0854	0.0278 - 0.169
SSS Thrill and Adventure Seeking	18.7	18.6	5.4 - 43	0.0960	0.0790	0.0238 - 0.162

Note: BIS-11 = Barratt Impulsivity Scale 11. SSS = Sensation Seeking Scale. CI = Confidence Interval. MVUE = Minimum variance unbiased estimate. Low MSE E = (Slightly biased) noncentrality estimate with lower total mean square error. η^2 = Eta-Squared, conventional definition of variance explained: SS(predictor variable)/SS(total). Ω^2 = Omega-Squared, adjusted, unbiased estimate of variance explained.

[1]Confidence interval for Low MSE E
[2]Confidence interval for η^2

Effect size estimates provided by the EFFECTSIZE model option of SAS proc GLM. See SAS documentation for SAS/STAT 14.1 for further details.

Information Classification: General
