# Supplementary material for: Brain Structural Features of Myotonic Dystrophy Type 1 and their Relationship with CTG Repeats
Source: J Neuromuscul Dis. Author manuscript; Available in PMC 2020 Sep 9. (PMC7480174; doi:10.3233/JND-190397)
Supplement: Supplementary Table 2 [file NIHMS1623355-supplement-Supplementary_Table_2.pdf]

Supplemental Table 2: Logistic Regression for All Personality Subscales.

Table 2

Logistic Regression Predicting Substance-Use Group

Subscale	Wald Chi-Square	Pr > Chi-Square
BIS-11		
Motor	5.41	0.2480
Attentional	6.58	0.1599
Nonplanning	8.68	0.0695
SSS		
Boredom Susceptibility	3.63	0.4583
Disinhibition[1]	24.4	<.0001
Experience Seeking[1]	3.87	0.4243
Thrill Adventure Seeking	11.6	0.0205

Note: BIS-11 = Barratt Impulsivity Scale 11. SSS = Sensation Seeking Scale.
[1]Substance use items removed.

Information Classification: General
