# Supplementary material for: Brain Structural Features of Myotonic Dystrophy Type 1 and their Relationship with CTG Repeats
Source: J Neuromuscul Dis. Author manuscript; Available in PMC 2020 Sep 9. (PMC7480174; doi:10.3233/JND-190397)
Supplement: Supplementary Table 1 [file NIHMS1623355-supplement-Supplementary_Table_1.pdf]

Supplemental Table 1. Between Group Analysis of Variance for All Personality Subscales.

Table 1

Personality Subscale Differences

Subscale	*F*	*p*	Control	sBinge	eBinge	MJ+Binge	MJ+eBinge
			M (SD)				
BIS-11							
Motor	6.94	.0001	20 (3.34)	21.7 (3.39)	23.3 (3.49)	23.1 (4.14)	23.5 (3.82)
Attentional	2.15	0.0754	15.2 (3.69)	15.3 (3.85)	15.8 (3.72)	16.8 (4.32)	17.5 (4.69)
Nonplanning	6.16	0.0001	19.9 (3.97)	22 (4.31)	23.2 (4.69)	24.1 (5.13)	24.5 (4.81)
SSS							
Boredom Susceptibility	3.34	0.0111	2.2 (1.57)	2.69 (1.89)	2.85 (1.79)	3.11 (1.91)	3.88 (2.44)
Disinhibition[1]	17.21	0.0001	2.55 (1.68)	3.74 (1.54)	4.29 (1.53)	5.17 (1.38)	4.8 (1.26)
Experience Seeking[1]	3.23	0.0135	4.35 (1.59)	4.35 (1.34)	5.07 (1.52)	5.29 (2.09)	4.96 (1.62)
Thrill Adventure Seeking	5.74	0.0002	6.85 (2.68)	7.23 (2.32)	8.73 (1.81)	8.23 (1.91)	7.48 (2.65)

Note: MJ = Marijuana. sBinge = Standard Binge. eBinge = Extreme Binge. BIS-11 = Barratt Impulsivity Scale 11. SSS = Sensation Seeking Scale.
[1]Substance use items removed

Information Classification: General
