# Supplementary material for: Brain Structural Features of Myotonic Dystrophy Type 1 and their Relationship with CTG Repeats
Source: J Neuromuscul Dis. Author manuscript; Available in PMC 2020 Sep 9. (PMC7480174; doi:10.3233/JND-190397)
Supplement: Supplementary Figure 1 [file NIHMS1623355-supplement-Supplementary_Figure_1.pdf]

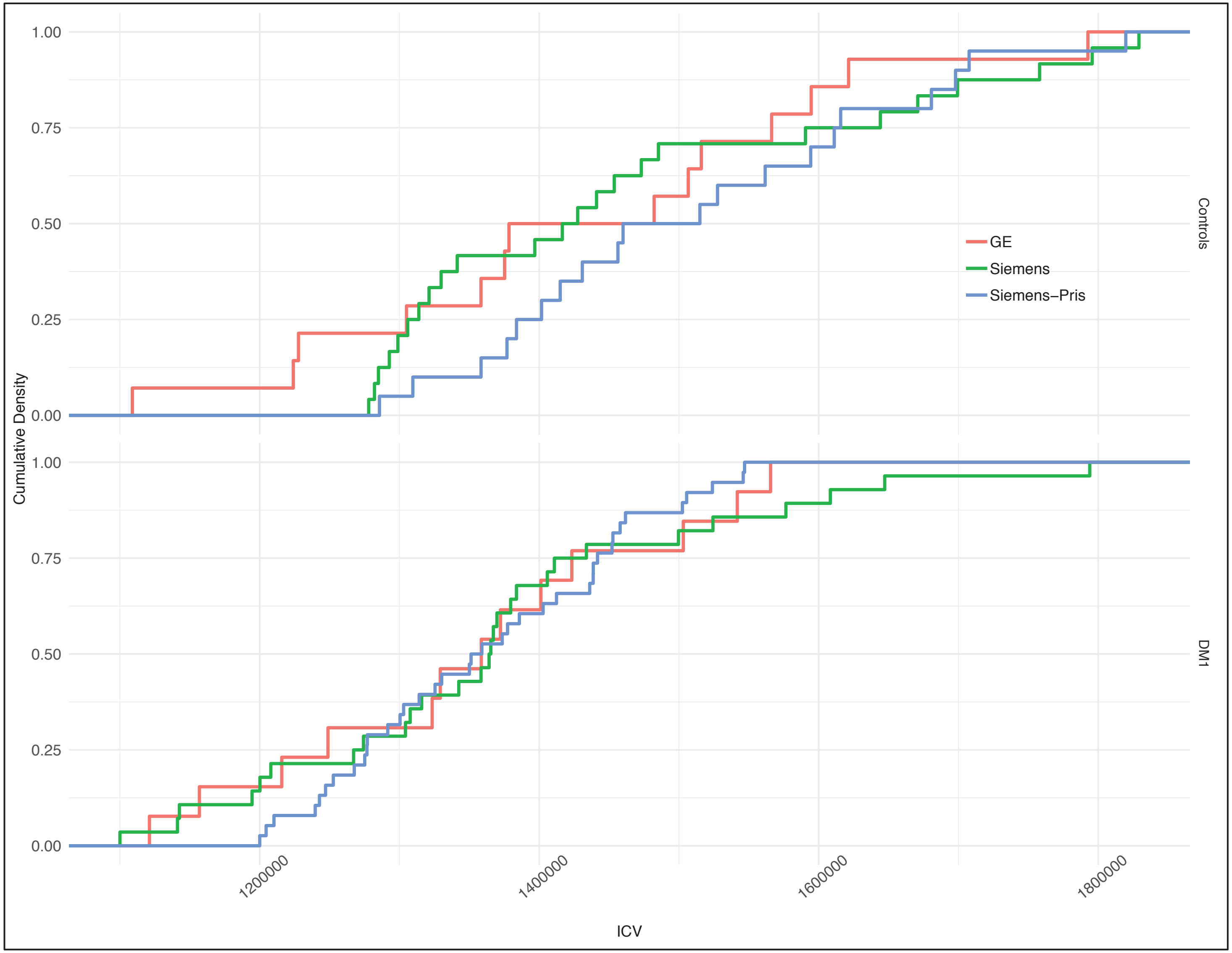

Supplementary Figure 1: Empirical cumulative distribution (y-axis) for each scanner (GE = red; Siemens=Green; Siemens-Pris=blue) for ICV (x-axis) shown separately for controls (top panel) and DM1 (bottom panel) following data harmonization.
