# Supplementary material for: Brain Structural Features of Myotonic Dystrophy Type 1 and their Relationship with CTG Repeats
Source: J Neuromuscul Dis. Author manuscript; Available in PMC 2020 Sep 9. (PMC7480174; doi:10.3233/JND-190397)

Supplementary Figure 2A to AB: Each panel (A through AB) shows the power-adjusted, standardized ROI on the y-axis as a function of standardized ICV on the x-axis. Individual observations are shown as circles and the regression line with confidence limits. All regression lines are horizontal, illustrating effective de-trending of ICV. pp = power-proportion

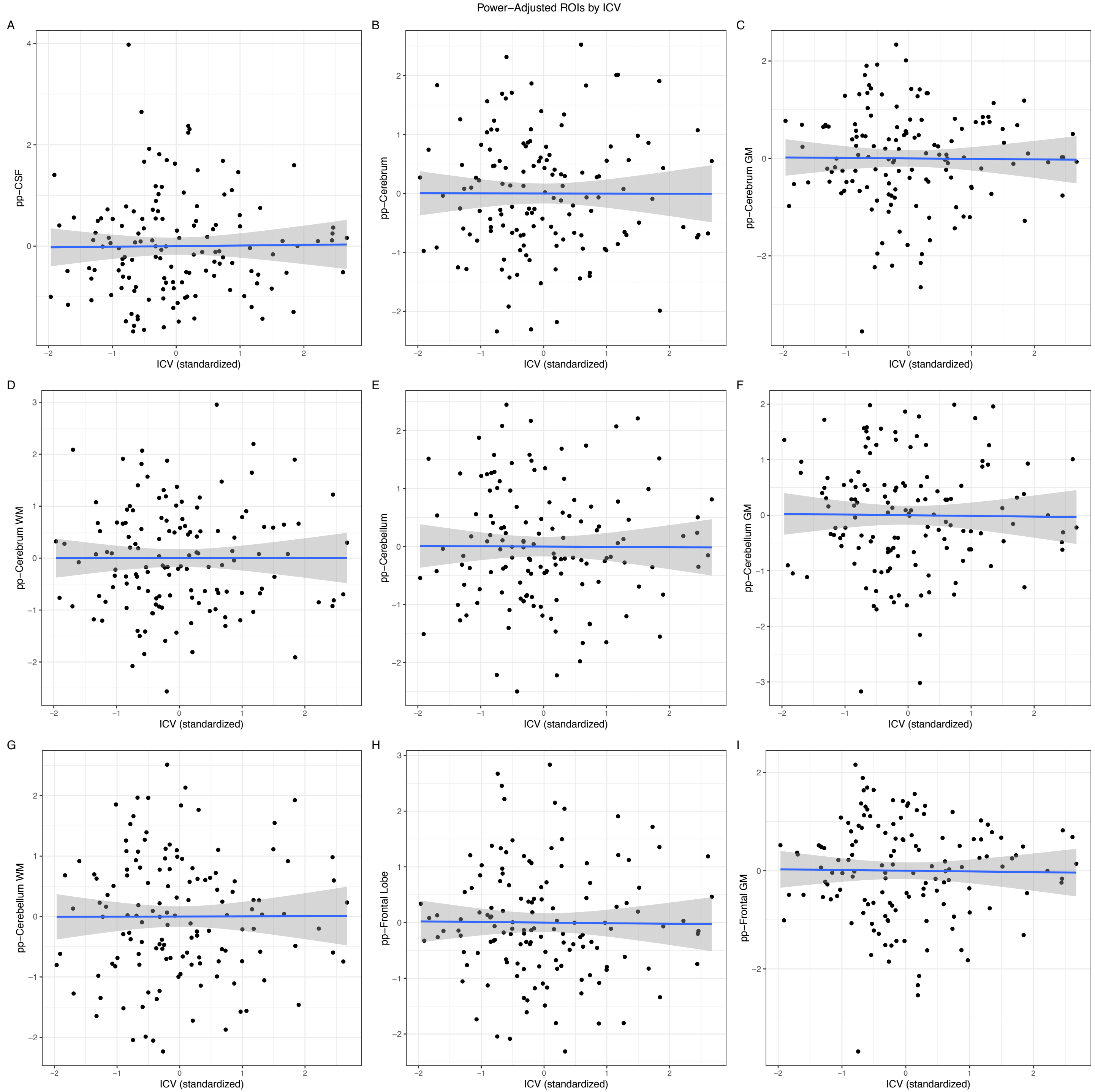

Power−Adjusted ROIs by ICV Continued

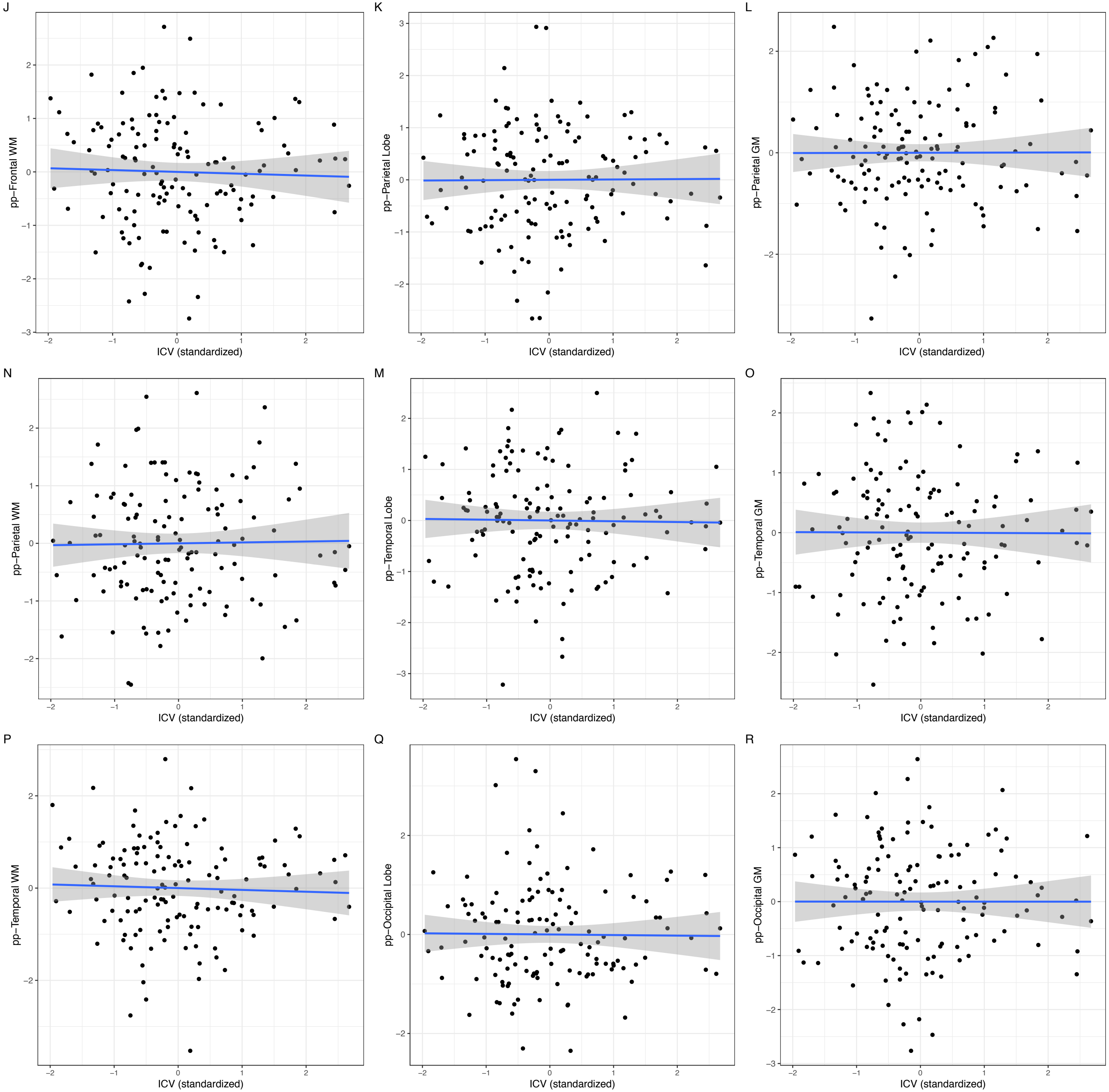

Power−Adjusted ROIs by ICV Continued

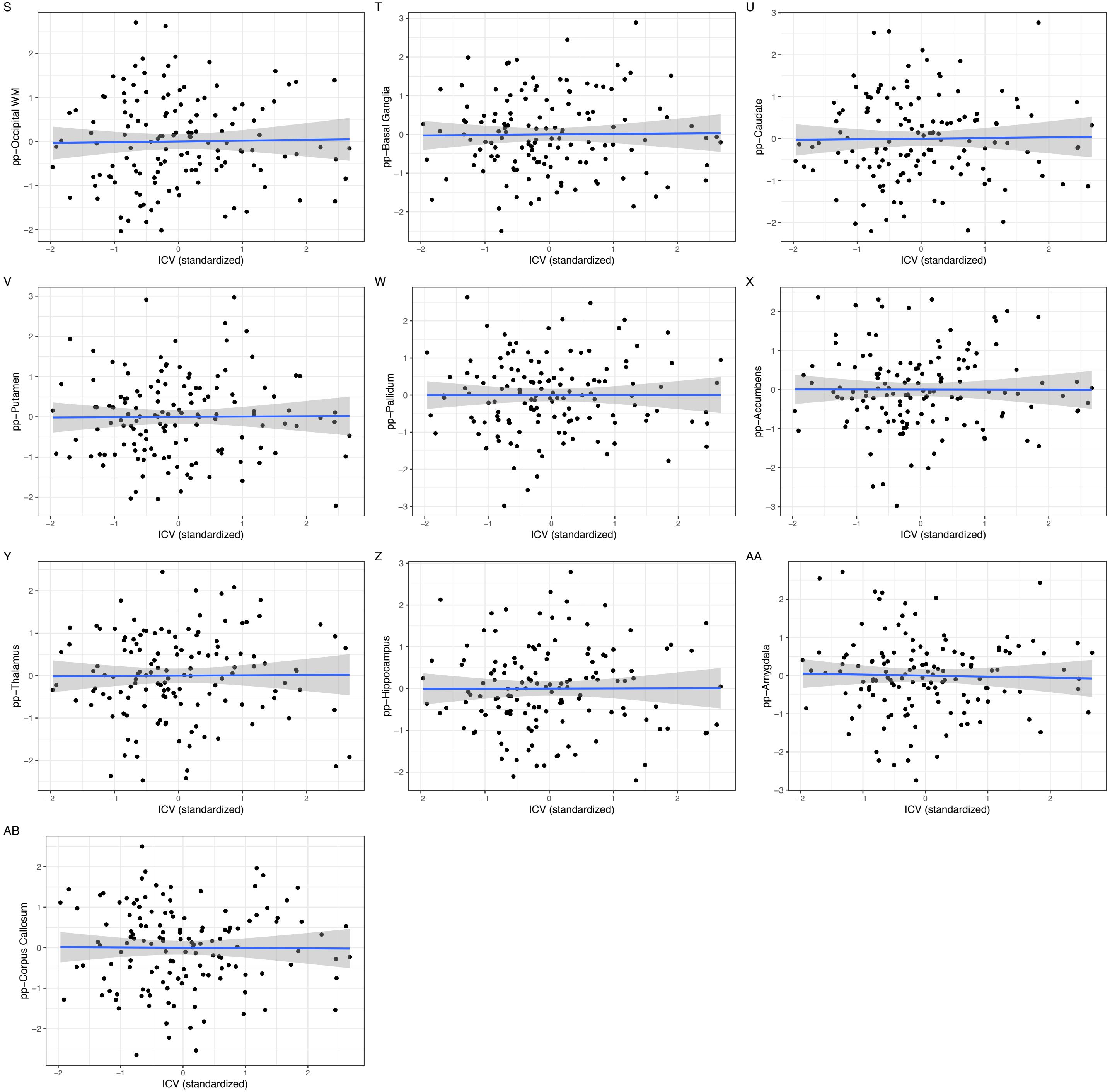

Supplement: Supplementary Figure 2 [file NIHMS1623355-supplement-Supplementary_Figure_2.pdf]
