# Supplementary material for: Brain Structural Features of Myotonic Dystrophy Type 1 and their Relationship with CTG Repeats
Source: J Neuromuscul Dis. Author manuscript; Available in PMC 2020 Sep 9. (PMC7480174; doi:10.3233/JND-190397)
Supplement: Supplementary Figure 3 [file NIHMS1623355-supplement-Supplementary_Figure_3.pdf]

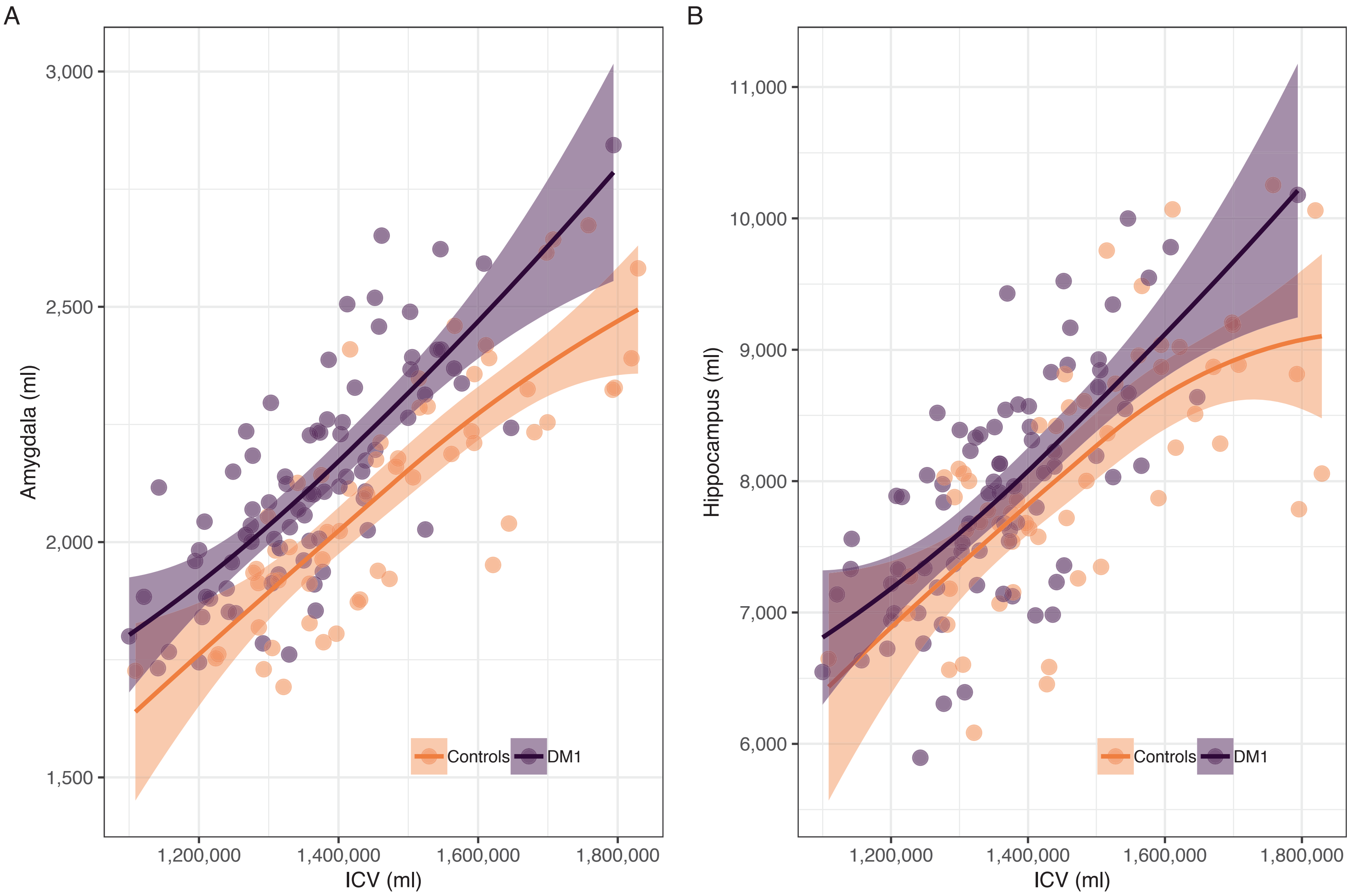

Supplementary Figure 3: Unadjusted amygdala volume (y-axis; Panel A) and hippocampus volume (y-axis; Panel B) as a function of ICV (x-axes). Controls are shown in orange and DM1 patients in purple. The DM1 group had larger unadjusted amygdala and hippocampal volume than did controls.
